# CT-based texture analysis potentially provides prognostic information complementary to interim fdg-pet for patients with hodgkin’s and aggressive non-hodgkin’s lymphomas

**DOI:** 10.1007/s00330-016-4470-8

**Published:** 2016-07-05

**Authors:** B. Ganeshan, K. A. Miles, S. Babikir, R. Shortman, A. Afaq, K. M. Ardeshna, A. M. Groves, I. Kayani

**Affiliations:** 1 Institute of Nuclear Medicine, University College London, Euston Rd, London, UK; 2 Human Health Division, Nuclear Medicine and Diagnostic Imaging Section, International Atomic Energy Agency (IAEA), Vienna, Austria

**Keywords:** Positron emission tomography and Computed tomography, Progression-free survival, Texture analysis, Risk stratification, Lymphoma

## Abstract

**Objectives:**

The purpose of this study was to investigate the ability of computed tomography texture analysis (CTTA) to provide additional prognostic information in patients with Hodgkin's lymphoma (HL) and high-grade non-Hodgkin's lymphoma (NHL).

**Methods:**

This retrospective, pilot-study approved by the IRB comprised 45 lymphoma patients undergoing routine 18F-FDG-PET-CT. Progression-free survival (PFS) was determined from clinical follow-up (mean-duration: 40 months; range: 10-62 months). Non-contrast-enhanced low-dose CT images were submitted to CTTA comprising image filtration to highlight features of different sizes followed by histogram-analysis using kurtosis. Prognostic value of CTTA was compared to PET FDG-uptake value, tumour-stage, tumour-bulk, lymphoma-type, treatment-regime, and interim FDG-PET (iPET) status using Kaplan-Meier analysis. Cox regression analysis determined the independence of significantly prognostic imaging and clinical features.

**Results:**

A total of 27 patients had aggressive NHL and 18 had HL. Mean PFS was 48.5 months. There was no significant difference in pre-treatment CTTA between the lymphoma sub-types. Kaplan-Meier analysis found pre-treatment CTTA (medium feature scale, p=0.010) and iPET status (p<0.001) to be significant predictors of PFS. Cox analysis revealed that an interaction between pre-treatment CTTA and iPET status was the only independent predictor of PFS (HR: 25.5, 95% CI: 5.4-120, p<0.001). Specifically, pre-treatment CTTA risk stratified patients with negative iPET.

**Conclusion:**

CTTA can potentially provide prognostic information complementary to iPET for patients with HL and aggressive NHL.

***Key Points*:**

• *CT texture-analysis (CTTA) provides prognostic information complementary to interim FDG-PET in Lymphoma.*

• *Pre-treatment CTTA and interim PET status were significant predictors of progression-free survival.*

• *Patients with negative interim PET could be further stratified by pre-treatment CTTA.*

• *Provide precision surveillance where additional imaging reserved for patients at greatest recurrence-risk.*

• *Assists in risk-adapted treatment strategy based on interim PET and CTTA.*

## Introduction

18F-Fluorodeoxyglucose (FDG) positron emission tomography (PET)–computed tomography (CT) is the standard imaging assessment at the end of treatment for patients with Hodgkin’s lymphoma (HL) and diffuse large B-Cell lymphoma (DLBCL) [1, 2], and is also recommended for initial staging. Additional interim PET (iPET) examinations are increasingly performed in clinical practice, typically after two cycles of treatment. The aims of iPET are to confirm the effectiveness of treatment and exclude progressive disease. Metabolic response demonstrated by PET-CT occurs earlier than anatomical response, and multiple studies have shown that iPET is a strong prognostic indicator in HL and aggressive non-Hodgkin’s lymphoma (NHL), outperforming the Prognostic Score and International Prognostic Index [3].

To date, the use of CT in lymphoma has been limited to the anatomical assessment of disease sites and, in conjunction with PET, for attenuation correction and localization of sites of tracer activity. However, studies in a range of other tumours have shown that, with appropriate quantitative image analysis, CT can also provide prognostic information [4–11]. One such analysis method is CT texture analysis (CTTA), which can be applied to images that are acquired in routine clinical practice, including the low-dose CT component of PET-CT [12]. The ability to obtain additional prognostic information from existing images is particularly pertinent for patients with lymphoma, many of whom are cured at a relatively young age and who therefore are exposed to the risk of developing a radiation-induced second cancer. Therefore, the aim of our study is to investigate the ability of CTTA applied to the low-dose CT component of pre-treatment PET-CT to provide additional prognostic information with specific reference to progression-free survival (PFS) in patients with Hodgkin’s and high-grade non-Hodgkin’s lymphoma, in comparison to FDG uptake on PET and other clinical markers.

## Materials and methods

### Patients

An institutional review board waiver was obtained for this retrospective study analysis since for this type of study formal ethical approval is not required. Informed consent was obtained from all individual participants included in the study. This single-institution pilot study used archived patient data previously obtained in routine diagnostic practice. Consecutive patients diagnosed with pathologically proven, newly diagnosed Hodgkin’s or high-grade non-Hodgkin’s lymphoma and treated at our institution from 2007 to 2013 were identified from the institutional lymphoma database. Inclusion criterion was that subjects were newly diagnosed Hodgkin’s or high-grade non-Hodgkin’s lymphoma patients who had a pre-treatment PET-CT scan performed before chemotherapy. Based on the above inclusion criterion, 45 patients were included in our study population (20 men and 25 women, mean age: 52, age range 22–81 years).

### Image acquisition

Following a 6-hour fast, patients received an intravenous injection of 370MBq FDG. A standard uptake period of 60 minutes after FDG injection was used. All imaging was performed on an integrated PET-CT scanner combining PET with a 64-slice multi-detector CT (VCT-XT Discovery, GE Healthcare, Amersham, UK). Low-radiation-dose CT without intravenous contrast agent for attenuation correction was performed from the skull base to upper thighs using the following parameters: 140 kV, 80 mAs, 0.8 s rotation time, pitch 1.5, 5-mm slice thickness, 5-mm collimation, FOV 400 mm, matrix size 512 x 512 and pixel resolution 0.98 mm x 0.98 mm. Axial CT slices were reconstructed with standard filter kernel and lung algorithms. The PET emission scan was obtained over the same anatomical area (vertex to mid thighs). All acquisitions were carried out in two-dimensional mode (2-D), consisting of an emission scan of 4 minutes per bed position. PET images were reconstructed using CT for attenuation correction by employing CT maps. Transaxial emission images of 5.47 × 5.47 × 3.27 mm (in plane matrix size 128 × 128) were reconstructed using ordered subsets expectation maximization (OSEM) with two iterations and 28 subsets. The axial field-of-view was 148.75 mm. The protocol of pre-treatment, interim (between 2-4 cycles of chemotherapy to assess response) and post-treatment (within 4 weeks following treatment to assess response) PET-CT imaging was performed using the above-described acquisition protocol for all the patients in our study. For the purpose of image analysis only the pre-treatment PET-CT was used to derive CTTA and PET FDG uptake.

### Image analysis (considering only the pre-treatment PET-CT scan)

Multi-parametric PET-CT image analysis comprised filtration-histogram-based CT texture analysis (CTTA—kurtosis at different spatial scale filters) and PET FDG uptake (maximum standardized uptake value: SUV_max_). Multiple lesions (up to five per patient, selected on the basis of most avid lesions on PET scan,) were analyzed for CTTA and PET FDG uptake for each patient. The average value of all the available lesions in a patient was considered as the representative CTTA and PET FDG uptake measurement for that particular patient. Each image data set was analyzed independently by operators blinded to the analysis results of the other data sets. Tumour regions of interest (ROIs) were drawn freehand, enclosing the lesion on the PET-CT displaying the axial image slice with the largest cross-section dimension of the lymphoma tumour using a standardized procedure so that ROIs were comparable in terms of anatomical location for all data sets, as described below.

#### FDG PET-CT

**Fig. 1:**
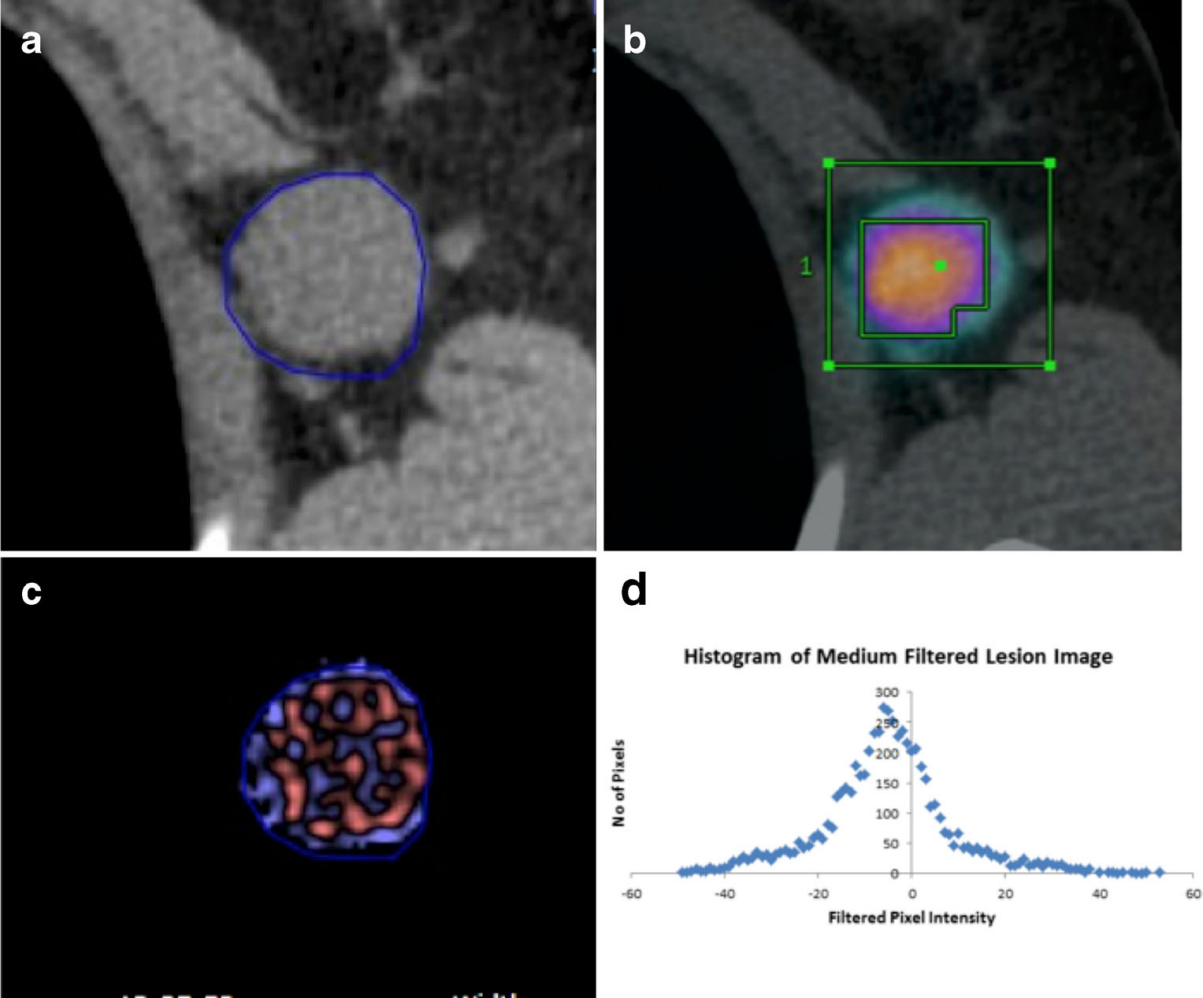
Multi-parametric PET-CT of non-Hodgkin lymphoma comprising CT image (**a**), fused FDG uptake on PET and CT image (**b**), CT texture map highlighting features at medium texture scale (**c**), and CT texture histogram (**d**)

The PET study and non-enhanced CT were viewed independently and as co-registered studies by a nuclear medicine physician (with > 10 years’ experience) using a commercial workstation (Advantage Windows 4.4, GE Healthcare). SUV_max,_ values for the entire tumour volume were obtained for each patient using an automated thresholding method (Fig. 1) [13, 14].

#### CT texture analysis (CTTA)

CT texture analysis (CTTA) was performed using TexRAD (TexRAD Ltd, www.texrad.com, Cambridge, UK part of Feedback Plc www.fbk.com), a proprietary commercial research software algorithm [15–17]. The texture within lymphoma tumours was assessed by a physician by constructing tumour ROIs on CT, under the supervision of an imaging researcher (with > 9 years’ of experience in CTTA) and a dual accredited Radiologist/Nuclear Medicine specialist (with > 10 years’ of PET-CT experience). Tumour ROIs constructed on the CT images were further refined by an automatic contouring procedure that excluded gas from the ROI by removing any pixels with attenuation values below -50 Hounsfield units (Fig. 1). Mean ROI size was 1890 pixels (range, 113-21175 pixels). CTTA comprised an image filtration-histogram approach where texture within the tumour ROI was quantified following Laplacian of Gaussian (LoG) band-pass (non-orthogonal) spatial-scale image filtration (SSF) to highlight features ranging from approximately 2mm (fine) to 6mm (coarse) in radius; 3–5mm in radius corresponds to medium-texture scales. Histogram analysis comprised quantifying kurtosis (*k*) of the tumour pixels with and without filtration (as a control). This parameter, summarized in Eq. 1, reflects peakedness and tailedness (in another term, the “sharpness”) of the histogram.

1 $$ \mathrm{k}=\frac{n\left(n+1\right){{\displaystyle \sum_{\left(x,y\right)\in R}\left[a\left(x,y\right)-\overline{a}\right]}}^4}{\left(n-1\right)\left(n-2\right)\left(n-3\right){\left[sd(a)\right]}^4}-3\frac{{\left(n-1\right)}^2}{\left(n-2\right)\left(n-3\right)} $$

Where

$$ \begin{array}{l}\overline{a}=\frac{1}{n}{\displaystyle \sum_{\left(x,y\right)\in R}\left[a\left(x,y\right)\right]}\\ {}sd={\left(\frac{1}{n-1}{{\displaystyle \sum_{\left(x,y\right)\in R}\left[a\left(x,y\right)-\overline{a}\right]}}^2\right)}^{\frac{1}{2}}\end{array} $$

*ā* is the mean value and *sd* is the standard-deviation within *R*, *R* is the ROI within the image *a(x,y)*, and *n* is the total number of pixels in *R*. The kurtosis value can be positive or negative. A positive kurtosis indicates a histogram that is more peaked than a Gaussian (normal) distribution. A negative kurtosis indicates that histogram is flatter than a Gaussian (normal) distribution. Filtration-histogram-based CT texture analysis makes the process of image-quantification intuitive to imaging practice (important for clinical-acceptance) and at the same-time, an “objective” way of quantifying heterogeneity. The filtration step extracts features of different sizes followed by histogram quantification. A recent article describes what the filtration-histogram technique of CTTA actually means in terms of image features [12]. In terms of image features, kurtosis is inversely related to the number of objects highlighted (whether bright or dark), and in some instances kurtosis is increased by intensity variations in highlighted objects. Thus, the combination of filtration-histogram (e.g. kurtosis) technique could reflect the three components of heterogeneity—objects/features of different sizes, numbers, and intensity variation in relation to the background/parenchyma of the tumour/tissue. Therefore, kurtosis post-filtration could be good enough to give an overall description of “heterogeneity”. Another reason for not looking at several other reported texture quantifications is the fact that looking at a large number of quantifications could lead to higher false discovery rate purely by chance because of multiple statistical tests involved in assessing individual parameter significance. Kurtosis post-filtration has also been shown to be associated with overall survival in other cancers such as colorectal and oesophageal cancers on CT [8, 9].

### Clinical parameters

Tumour stage (Ann Arbor), bulk (sum of individual lesion areas expressed as number of pixels), type of lymphoma, treatment (standard or non-standard chemotherapy regime), and iPET findings were derived to further assess the ability of these clinical parameters to predict progression-free survival (PFS).

Standard chemotherapy was defined as R-CHOP 21 (rituximab–cyclophosphamide, doxorubicin, vincristine, and prednisolone) for diffuse, large B-cell lymphoma (DLBCL), ABVD (doxorubicin, bleomycin, vinblastine, dacarbazine) for Hodgkin’s lymphoma. Standard chemotherapy for Burkitt’s lymphoma was defined as R-CODOX (rituximab–cyclophosphamide, vincristine, doxorubicin, and methotrexate)/M-IVAC (etopisde, ifosamide, and cytarabine). Standard chemotherapy for T-cell lymphoma was R-CHOP. Of the 45 patients, 11 of them (DLBCL, n=4, Burkitt’s, n=1, T-cell lymphoma, n=1, and Hodgkin’s, n=5) had additional treatment with radiotherapy.

iPET (after 2-4 cycles of chemotherapy) status was based on assessment by the reporting physician and subsequent review by a nuclear medicine physician (with >10 years’ experience) within a multi-disciplinary team (MDT) setting. A score of 4 or higher on the Deauville scoring system was taken as positive, whereas a score of 1–3 was taken as negative on iPET [18].

### Follow-up

The average follow-up period was 40 (10-62) months from the pre-treatment PET-CT. Progression-free survival (PFS) was defined as the time between the date of the pre-treatment PET-CT and the date of the last clinical follow-up (for patients in remission) or the date of relapse/progression. All the relevant clinical data, follow-up, and survival data were obtained by a specialist cancer research nurse (with > 6 years’ experience).

### Statistical analysis

For each patient, average CTTA and PET FDG uptake measurements from all the lesions on the pre-treatment CT and PET were employed for the statistical analyses. For all statistical tests, a p-value less than 0.05 was considered to be significant. A non-parametric Mann–Whitney test evaluated the difference in the above pre-treatment PET-CT imaging markers between the lymphoma sub-types (Hodgkin’s and high-grade non-Hodgkin’s lymphoma). To identify the best pre-treatment CTTA parameter (to undergo survival-analysis), a Mann–Whitney test assessed the difference in texture between patients who relapsed from patients who did not relapse. Additionally, a Mann–Whitney test also assessed the difference in PET SUV_max_ between patients who relapsed from patients who did not relapse.

Univariate optimized Kaplan-Meier analysis assessed the ability of pre-treatment imaging (i.e., best CTTA, PET-FDG uptake) and clinical markers (i.e., tumour-stage, bulk, lymphoma type, treatment, and iPET status) to predict PFS. Differences between the Kaplan Meier survival curves were evaluated by a non-parametric log–rank test. An optimal threshold was identified for each marker, which best separated (lowest p value from log–rank test) the good and poor prognostic patient sub-groups. Additionally, to address the multiple survival comparisons (n=7) arising from the above step, a Benjamini-Hochberg correction was employed to control the false discovery rate at 0.05. Multivariate Cox regression analysis was undertaken by developing a model incorporating all the significant (univariate) markers to determine which parameters and/or interactions between parameters were independent predictors of PFS. Statistical analyses were performed using IBM SPSS Statistics for Windows (version 19.0, IBM Corp. Armonk, NY).

## Results

In total, 27 out of 45 patients had high-grade non-Hodgkin’s lymphoma (DLBCL: n=19, Burkitt's: n=6, T-cell: n=2) and the remaining 18 had Hodgkin’s lymphoma. The patient cohort comprised five patients with stage I, 17 with stage II, nine with stage III, and 14 with stage IV tumours. Patients were treated with standard (n=35) or non-standard (n=10) chemo-therapy regimes.

In all, 37/45 patients had an iPET, seven of which were positive (NHL=4 and HL=3) and 30 of which were negative (NHL, n=19 and HL, n=11). Subsequently, clinical and imaging follow-up identified that 12 of 45 patients had progressed on first-line treatment. The mean (95 % confidence interval) PFS was 48.5 (41.9-55.2) months.

A total of 151 (non-Hodgkin’s lymphoma, n=86 and Hodgkin’s lymphoma, n=65) target lesions were analyzed for pre-treatment CTTA and PET FDG uptake. Mean and standard-deviation for all the imaging (CTTA and PET FDG uptake) parameters are summarized in Table 1.

**Table 1:** Mean and standard-deviation (SD) of all the imaging parameters employed in the study

Tumour characteristic	Mean	SD
CTTA (Kurtosis)		
Without-filtration	2.77	7.95
Fine (SSF=2 mm radius)	1.01	1.72
Medium (SSF=3 mm radius)	1.23	1.86
Medium (SSF=4 mm radius)	1.22	1.80
Medium (SSF=5 mm radius)	1.18	1.82
Coarse (SSF=6 mm radius)	1.01	1.98
PET (FDG-uptake)		
*SUV*_*max*_	22.14	16.77

### Difference in pre-treatment PET-CT imaging markers between Hodgkin’s and high-grade non-Hodgkin’s Lymphoma

There was no significant difference for the different pre-treatment CTTA measures between the lymphoma sub-types (Hodgkin’s and high-grade non-Hodgkin’s lymphoma), but they are generally found to be higher in the high-grade NHL compared to in HL. However, pre-treatment PET SUV_max_ was significantly different and higher in the high-grade NHL compared to in HL (median SUV_max_ was 20.5 vs 13.2, p=0.002).

### Difference in pre-treatment PET-CT imaging markers between relapse and no relapse

There was no significant difference for the different pre-treatment PET-CT measures between the patients who relapsed and those who did not relapse, as indicated in Table 2. Amongst the different CTTA measures, Kurtosis at medium-texture at SSF=5mm was the best metric in terms of differentiating between relapse and no relapse (p=0.053) and was chosen for survival analysis.

**Table 2:** Summary of the median values of the pre-treatment PET-CT imaging markers within the lymphoma patients who relapsed and did not relapse, and the corresponding p-values from Mann–Whitney test

Tumour characteristic	Median		p-value (Mann Whitney)
	Relapse	No relapse	
CTTA (Kurtosis)			
Without filtration	1.28	0.58	0.170
Fine (SSF=2 mm radius)	0.68	0.45	0.281
Medium (SSF=3 mm radius)	1.28	0.47	0.186
Medium (SSF=4 mm radius)	0.86	0.33	0.091
Medium (SSF=5 mm radius)	1.13	0.16	0.053
Coarse (SSF=6 mm radius)	1.35	0.01	0.111
PET (FDG-uptake)			
*SUV*_*max*_	20.4	17.2	0.178

### Univariate Kaplan-Meier analysis

**Fig. 2:**
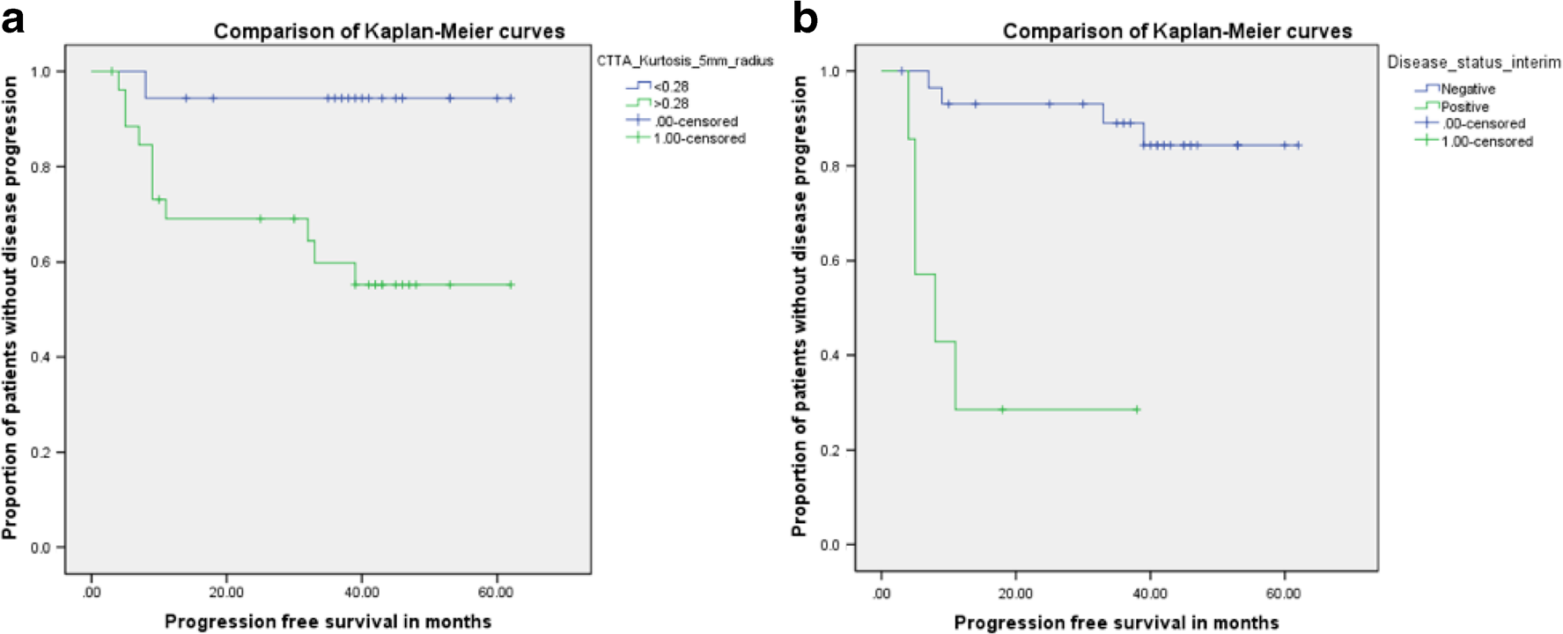
Kaplan Meier curves showing the proportion of patients without disease progression for (**a**) CTTA (Kurtosis at medium-texture SSF=5mm radius, p=0.010) and (**b**) status at the interim PET (p<0.001)

The ability of each imaging and clinical parameter to predict PFS at the optimal threshold is summarized in Table 5. After applying a Benjamini-Hochberg correction, only two parameters were significant from the univariate analysis. Pre-treatment imaging marker (CTTA: Kurtosis at medium-texture—SSF=5mm, p=0.010, Table 3, Fig. 2a) and iPET findings (p<0.001, Table 3, Fig. 2b) were the only predictors of PFS. Baseline PET FDG uptake (SUV_max_), lymphoma type, bulk, stage, and treatment were not significant predictors of PFS (Table 3).

**Table 3:** Summary of univariate Kaplan-Meier survival analysis for each imaging and clinical markers in order of significance (lowest p value from log-rank test first), and the corresponding Benjamini-Hochberg corrected p values. P-values highlighted in bold are significant after the correction

Tumour characteristic	Optimal threshold	Mean survival in months (number of patients)		p – value (KM)	Corrected p value
		Above threshold	Below threshold		
iPET status (0 – negative & 1 – positive)	>0	15.6 (7)	56.0 (30)	<0.001	0.007
CTTA (Kurtosis)					
Medium (SSF=5 mm radius)	>0.28	41.3 (27)	59.0 (18)	0.010	0.014
PET (FDG-uptake)					
*SUV*_*max*_	>12.36	45.0 (36)	- (9)	0.051	0.021
Type (0 – Hodgkin & 1 – non-Hodgkin)	>0	44.0 (27)	56.1 (18)	0.060	0.029
Stage	>II	38.0 (23)	53.9 (22)	0.188	0.036
Treatment (0 – non-standard & 1 – standard)	>0	46.3 (35)	44.0 (10)	0.211	0.043
Bulk (number of pixels)	>18823	38.6 (23)	40.8 (22)	0.531	0.050

### Multivariate Cox regression analysis

**Fig. 3:**
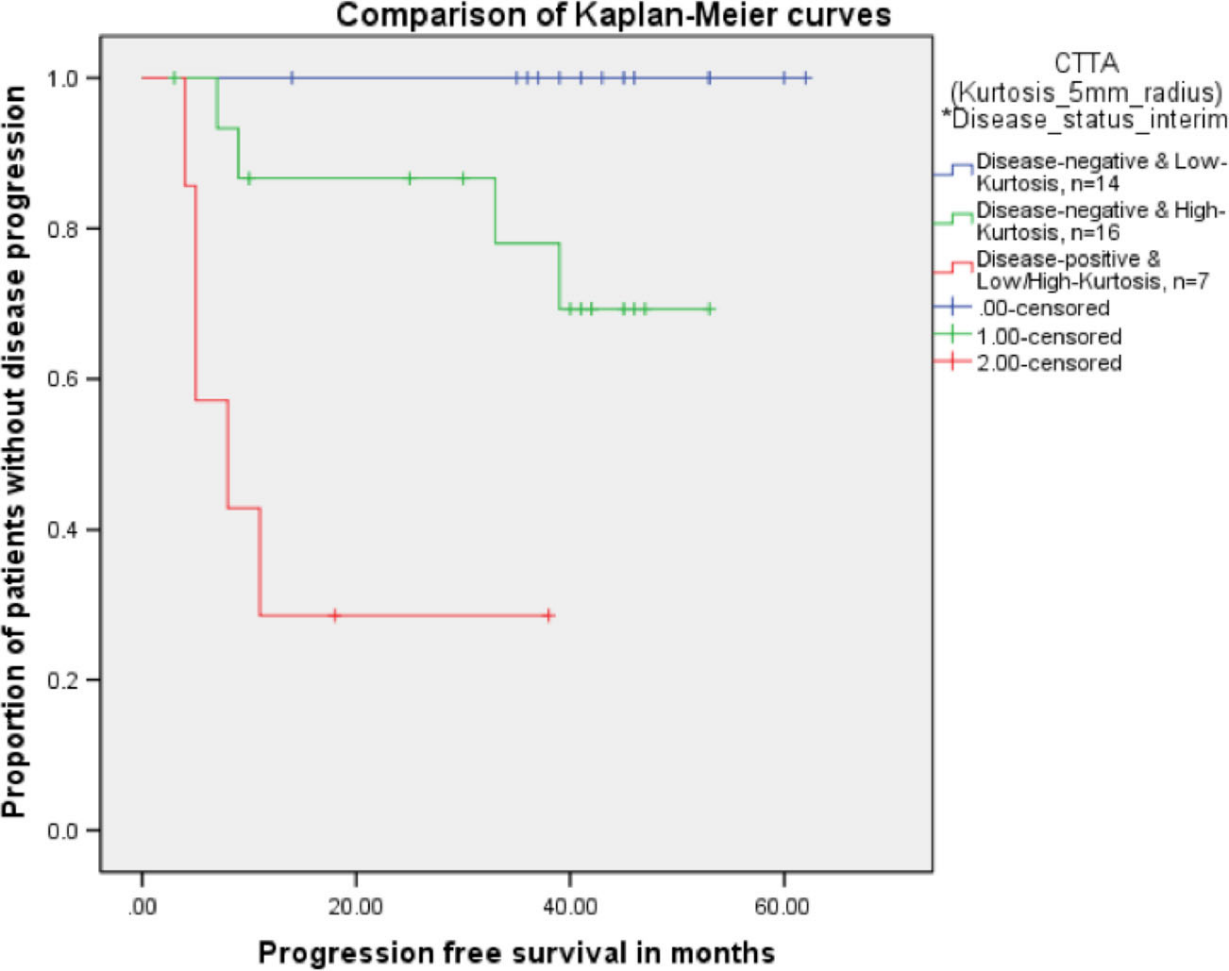
Kaplan Meier curves showing the proportion of patients without disease progression for the best parameter from the multivariate Cox regression analysis, i.e., CTTA (Kurtosis at medium-texture SSF=5mm radius)* status at the interim PET (p<0.001)

Including only parameters significantly prognostic on univariate analysis (pre-treatment CTTA, medium Kurtosis, and iPET status) along with their interactions, Cox regression analysis indicated that a combination of pre-treatment CTTA and iPET status was the only independent predictor of PFS (hazard ratio, HR=25.5, 95 % CI=5.4 – 120, p<0.001). Specifically this combination was able to identify among patients with negative iPET (n=30) two sub-groups: a sub-group (n=14) with lower kurtosis values at CTTA (mean (range) : -0.16 (-0.86 - 0.28)) experienced longer mean PFS (59.0 months, Fig. 3) while the other sub-group (n=16) demonstrated higher kurtosis values at CTTA (mean (range) : 2.18 (0.32 - 6.41)) and shorter mean PFS (41.3 months, Fig. 3). CTTA was not discriminatory for patients with positive findings at interim scan. These patients recorded the highest kurtosis values at CTTA (mean (range): 2.40 (0.72 - 4.59)) and the shortest mean PFS (7.3 months, Fig. 3). Furthermore, no relapses were observed in the lower-risk group within the 40-month mean follow-up period compared to a relapse rate of 31 % in the intermediate risk group and 71 % in the higher risk group (i.e., for patients with positive iPET).

## Discussion

The results from our pilot study demonstrate a potential association between pre-treatment CTTA values from non-contrast enhanced tumour images and progression-free survival (PFS) in patients with HL and aggressive NHL. Pre-treatment CTTA was shown to be a significant predictor of PFS. The other clinical markers (pre-treatment SUV, lymphoma type, stage, and treatment) were not found to be significant predictors of PFS, perhaps owing to the small size of the study population. Our study also indicates the potential for baseline CTTA on pre-treatment staging scan to complement the prognostic value of iPET, as the only independent predictor of PFS on multivariate analysis was a combination of pre-treatment CTTA and iPET status. Pre-treatment CTTA was able to further stratify patients based on their iPET status. Those patients with a negative iPET who had a beneficial pre-treatment CTTA had no relapses, whereas those with a negative iPET who had adverse baseline CTTA had higher relapse rates despite achieving negative iPET status.

Many studies have confirmed the importance of FDG-PET and PET-CT for staging and response assessment in lymphoma [1–3, 18–24]. Recently, a few studies have highlighted the potential of texture analysis in lymphoma for computer-aided staging/diagnosis and as a treatment response marker using the CT component of PET-CT, contrast-enhanced CT, and magnetic resonance imaging (MRI); however, data is very limited [25–27]. The study in pre- and post-chemotherapy on contrast-enhanced CT in patients with HL demonstrated the ability of texture features combined with classical CT parameters to be potentially useful in identifying complete responders [27]. Our pilot study focused on using the low-dose CT (component of PET-CT) texture analysis in HL and aggressive NHL lesions as a prognostic marker assessing progression-free survival.

There have been no studies reporting pathological/biological correlates for CTTA in lymphoma, an important aspect as part of validating imaging biomarkers. One possible correlate for CTTA is fibrosis, which has been shown to be a significant component of lymphoma with variable patterns of fibrosis described between lymphoma sub-types [28]. A recent study has suggested that fibrosis within lymphoma may be related to treatment response [29]. Sieren et al [30] have further shown a correlation between CT Hounsfield units (HU) and histology, particularly higher HU on CT is associated with fibrosis in lung cancer. It is feasible that CTTA evaluates attenuation variations resulting from different patterns of fibrosis. Computer simulations have characterized the relationship between CT image features and the texture parameter used in our study (kurtosis) [12]. The finding that this parameter is associated with shorter PFS in our study of lymphoma patients is concordant with studies that have shown the same CTTA parameter to be significantly associated with survival in colorectal and oesophageal cancers [8, 9]. Texture quantified as kurtosis has also shown to benefit from relative insensitivity to variations in CT acquisition parameters [31] and favorable test-retest performance on MRI [32].

Limitations of our preliminary study include the relatively small sample size of the patient population and mixed nature of lymphoma subtypes (Hodgkin’s and aggressive non-Hodgkin’s lymphoma). However, combining these lymphoma sub-types can be justified by that fact that there was no significant difference in CTTA values between Hodgkin’s and aggressive non-Hodgkin’s lymphoma in our study.

We did not assess texture on interim CT scans for two reasons. Firstly, not all patients had interim PET scans. Also a large proportion of iPET scans were negative and, in many of these, the nodal disease had resolved or was, using the low-dose CT, impossible to reliably delineate with CT. Early treatment changes in tumour texture, however, provides another potential useful and important response marker requiring evaluation in further, larger studies.

It was not possible to assess whether baseline CTTA was able to predict, as it could in those with negative iPET, an outcome in patients with positive iPET scans. There were a small number of patients with positive iPET who had uniformly poor outcomes, which precluded this analysis. As iPET has low or variable reported positive predictive value in DLBCL, this would be useful to investigate in future studies. Quantification of changes in tumour FDG using % reduction in SUV_max_ values or other functional markers such as total lesion glycolysis and metabolic tumour volume may be necessary to optimize the prognostic performance of iPET, particularly in DLBCL [33–35], and may help reduce false-positive findings relative to visual assessment alone. This was not assessed in our study, as uptake times for all scans were not strictly controlled.

Our pilot data merits further evaluation of the utility of CTTA in larger multi-center studies. Incorporation within existing and future randomized clinical trials focused on risk-adapted treatment strategy based on iPET-CT would further be a potentially attractive approach. Future studies could also assess the complementary value of CTTA and iPET in different types of lymphoma as well as evaluate CTTA values between baseline and interim PET examinations.

## Conclusion

In conclusion, this pilot study has demonstrated that CT-based texture analysis can potentially provide prognostic information that is complementary to interim FDG-PET for patients with Hodgkin’s and aggressive non-Hodgkin’s lymphomas. By analyzing the low-dose CT component of PET-CT acquired in routine clinical practice, CTTA entails no additional radiation exposure and can be readily incorporated into clinical workflows.

## References

[1] Gallamini A, Borra A (2014) Role of PET in Lymphoma. Curr Treat Options Oncol. [Epub ahead of print] 10.1007/s11864-014-0278-4 24619427

[2] Kostakoglu L, Cheson BD (2014). Current role of FDG PET/CT in lymphoma. Eur J Nucl Med Mol Imaging.

[3] Barrington SF, Mikhaeel NG, Kostakoglu L (2014). Role of imaging in the staging and response assessment of lymphoma: consensus of the international conference of malignant lymphomas imaging working group. J Clin Oncol.

[4] Davnall F, Yip CS, Ljungqvist G, Selmi M, Ng F, Sanghera B, Ganeshan B, Miles KA, Cook GJ, Goh V (2012). Assessment of tumor heterogeneity: an emerging imaging tool for clinical practice?. Insights Imaging.

[5] Ganeshan B, Miles KA (2013). Quantifying tumour heterogeneity with CT. Cancer Imaging.

[6] Win T, Miles KA, Janes SM, Ganeshan B, Shastry M, Endozo R, Meagher M, Shortman RI, Wan S, Kayani I, Ell PJ, Groves AM (2013). Tumor Heterogeneity and Permeability as Measured on the CT Component of PET/CT Predict Survival in Patients with Non-Small Cell Lung Cancer. Clin Cancer Res.

[7] Ganeshan B, Skogen K, Pressney I, Coutroubis D, Miles K (2012). Tumour heterogeneity in oesophageal cancer assessed by CT texture analysis: preliminary evidence of an association with tumour metabolism, stage, and survival. Clin Radiol.

[8] Ng F, Ganeshan B, Kozarski R, Miles KA, Goh V (2013). Assessment of primary colorectal cancer heterogeneity by using whole-tumor texture analysis: contrast-enhanced CT texture as a biomarker of 5-year survival. Radiology.

[9] Yip C, Landau D, Kozarski R, Ganeshan B, Thomas R, Michaelidou A, Goh V (2014). Primary esophageal cancer: heterogeneity as potential prognostic biomarker in patients treated with definitive chemotherapy and radiation therapy. Radiology.

[10] Zhang H, Graham CM, Elci O, Griswold ME, Zhang X, Khan MA, Pitman K, Caudell JJ, Hamilton RD, Ganeshan B, Smith AD (2013). Locally advanced squamous cell carcinoma of the head and neck: CT texture and histogram analysis allow independent prediction of overall survival in patients treated with induction chemotherapy. Radiology.

[11] Goh V, Ganeshan B, Nathan P, Juttla JK, Vinayan A, Miles KA (2011). Assessment of response to tyrosine kinase inhibitors in metastatic renal cell cancer: CT texture as a predictive biomarker. Radiology.

[12] Miles KA, Ganeshan B, Hayball MP (2013). CT texture analysis using the filtration-histogram method: what do the measurements mean?. Cancer Imaging.

[13] Groves AM, Shastry M, Rodriguez-Justo M (2011). 18F-FDG PET and biomarkers for tumor angiogenesis in early breast cancer. Eur J Nucl Med Mol Imaging.

[14] Erdi YE, Rosenzweig K, Erdi AK (2002). Radiotherapy treatment planning for patients with non-small cell lung cancer using positron emission tomography (PET). Radiother Oncol.

[15] Ganeshan B, Mandeville H, Burke M, Bell A, Townsend E, Hoskin P, Miles KA, Goh V (2013). CT of Non-small cell lung cancer (NSCLC): Histopathological correlates for texture parameters. Radiology.

[16] Ganeshan B, Panayiotou E, Burnand K, Dizdarevic S, Miles K (2012). Tumour heterogeneity in non-small cell lung carcinoma assessed by CT texture analysis: a potential marker of survival. Eur Radiol.

[17] Ganeshan B, Abaleke SC, Young RCD, Chatwin CR, Miles KA (2010). Texture analysis of non-small cell lung cancer on unenhanced computed tomography: Initial evidence for a relationship with tumor glucose metabolism and stage. Cancer Imaging.

[18] Gallamini A, Barrington SF, Biggi A (2014). The predictive role of interim Positron Emission Tomography on Hodgkin lymphoma treatment outcome is confirmed using the 5-point scale interpretation criteria. Haematologica.

[19] Podoloff DA, Macapinlac HA (2007). PET and PET/CT in Management of the Lymphomas. Radiol Clin N Am.

[20] Allen-Auerbach M, De Vos S, Czernin J (2008). The Impact of Fluorodeoxyglucose Positron Emission Tomography in Primary Staging and Patient Management in Lymphoma Patients.’. Radiol Clin N Am.

[21] Avril NE, Weber WA (2005). Monitoring response to treatment in patients utilizing PET. Radiol Clin N Am.

[22] Tatsumi M, Cohade C, Nakamoto Y, Fishman EK, Wahl RL (2005). Direct Comparison of FDG PET and CT Findings in Patients with Lymphoma: Initial Experience. Radiology.

[23] Hampson FA, Shaw AS (2008). Response assessment in lymphoma. Clin Radiol.

[24] An YS, Yoon JK, Lee SJ, Jeong SH, Lee HW (2016) Clinical significance of post-treatment (18)F-fluorodeoxyglucose uptake in cervical lymph nodes in patients with diffuse large B-cell lymphoma. Eur Radiol 10.1007/s00330-016-4365-8 27193777

[25] Lartizien C, Rogez M, Niaf E, Ricard F (2014). Computer aided staging of lymphoma patients with FDG PET/CT imaging based on textural information. IEEE J Biomed Health Inform.

[26] Harrison LC, Luukkaala T, Pertovaara H et al (2009) Non-Hodgkin lymphoma response evaluation with MRI texture classification. J Exp Clin Cancer Res 28:87 10.1186/1756-9966-28-87 PMC2711966 19545438

[27] Knogler T, El-Rabadi K, Weber M, Karanikas G, Mayerhoefer ME (2014). Three-dimensional texture analysis of contrast-enhanced CT images for treatment response assessment in Hodgkin lymphoma: comparison with F-18-FDG PET. Med Phys.

[28] Tataroglu C, Sarioglu S, Kargi A, Ozkal S, Aydin O (2007). Fibrosis in Hodgkin and non-Hodgkin lymphomas. Pathol Res Pract.

[29] Lenz G, Wright G, Dave SS (2008). Stromal Gene Signatures in Large-B-Cell Lymphomas. Lymphoma/Leukemia Molecular Profiling Project. N Engl J Med.

[30] Sieren JC, Smith AR, Thiesse J (2011). Exploration of the volumetric composition of human lung cancer nodules in correlated histopathology and computed tomography. Lung Cancer.

[31] Miles KA, Ganeshan B, Griffiths MR, Young RC, Chatwin CR (2009). Colorectal cancer: texture analysis of portal phase hepatic CT images as a potential marker of survival. Radiology.

[32] Gourtsoyianni S, Ljungqvist G, Khan A, et al. (2013) Reproducibility of MR texture analysis in primary rectal cancer. In European Society of Radiology, Vienna, Austria

[33] Mhlanga JC, Durand D, Tsai HL (2014). Differentiation of HIV-associated lymphoma from HIV-associated reactive adenopathy using quantitative FDG PET and symmetry. Eur J Nucl Med Mol Imaging.

[34] Gallicchio R, Mansueto G, Simeon V (2014). F-18 FDG PET/CT quantization parameters as predictors of outcome in patients with diffuse large B-cell lymphoma. Eur J Haematol.

[35] Kostakoglu L, Cheson BD, Casasnovas RO (2011). SUV_max_ reduction improves early prognosis value of interim positron emission tomography scans in diffuse large B-cell lymphoma. Blood.

